# Optimization of construct design and fermentation strategy for the production of bioactive ATF-SAP, a saporin based anti-tumoral uPAR-targeted chimera

**DOI:** 10.1186/s12934-016-0589-1

**Published:** 2016-11-14

**Authors:** Alfredo Errico Provenzano, Riccardo Posteri, Francesco Giansanti, Francesco Angelucci, Sopsamorn U. Flavell, David J. Flavell, Maria Serena Fabbrini, Danilo Porro, Rodolfo Ippoliti, Aldo Ceriotti, Paola Branduardi, Riccardo Vago

**Affiliations:** 1 Istituto Biologia e Biotecnologia Agraria, CNR, Milan, Italy; 2 Dipartimento di Biotecnologie e Bioscienze, Università degli Studi di Milano-Bicocca, Milan, Italy; 3 Department of Life, Health and Environmental Sciences, University of L’Aquila, L’Aquila, Italy; 4 Interuniversity Consortium INBB Biostructures and Biosystems National Institute, Rome, Italy; 5 The Simon Flavell Leukaemia Research Laboratory, (Leukaemia Busters), Southampton General Hospital, Southampton, UK; 6 Urological Research Institute, Division of Experimental Oncology, IRCCS San Raffaele Hospital, Milan, Italy; 7 Università Vita-Salute San Raffaele, Milan, Italy

**Keywords:** Targeted therapy, Saporin, Yeast expression system, Fed-batch production, Chimeric fusions, Ribosome inactivating proteins

## Abstract

**Background:**

The big challenge in any anti-tumor therapeutic approach is represented by the development of drugs selectively acting on the target with limited side effects, that exploit the unique characteristics of malignant cells. The urokinase (urokinase-type plasminogen activator, uPA) and its receptor uPAR have been identified as preferential target candidates since they play a key role in the evolution of neoplasms and are associated with neoplasm aggressiveness and poor clinical outcome in several different tumor types.

**Results:**

To selectively target uPAR over-expressing cancer cells, we prepared a set of chimeric proteins (ATF-SAP) formed by the human amino terminal fragments (ATF) of uPA and the plant ribosome inactivating protein saporin (SAP). Codon-usage optimization was used to increase the expression levels of the chimera in the methylotrophic yeast *Pichia pastoris*. We then moved the bioprocess to bioreactors and demonstrated that the fed-batch production of the recombinant protein can be successfully achieved, obtaining homogeneous discrete batches of the desired constructs. We also determined the cytotoxic activity of the obtained batch of ATF-SAP which was specifically cytotoxic for U937 leukemia cells, while another construct containing a catalytically inactive mutant form of SAP showed no activity.

**Conclusion:**

Our results demonstrate that the uPAR-targeted, saporin-based recombinant fusion ATF-SAP can be produced in a fed-batch fermentation with full retention of the molecules selective cytotoxicity and hence therapeutic potential.

**Electronic supplementary material:**

The online version of this article (doi:10.1186/s12934-016-0589-1) contains supplementary material, which is available to authorized users.

## Background

Cancer is a leading cause of death accounting for one-third of deaths worldwide. Until recently, conventional anticancer chemo- and/or radiotherapy have had limited effectiveness, with significant life threatening side effects and proving non-curative in a majority of cases. More efficient therapeutic strategies with less side effects are therefore urgently needed. Recent progress in the treatment of cancer have come from approaches where only the cancer cell is targeted leaving normal tissues in the patients body unharmed thus reducing some of the life threatening side effects associated with conventional chemo/radiotherapy. This has opened up the possibility of effective and personalized treatment based on the molecular profile of the tumor cells. Various molecules over-expressed on the surface of cancer cells have been discovered and exploited to design targeted drugs.

Ribosome-inactivating proteins (RIPs) belong to the N-glycosidase family of toxins capable of specifically and irreversibly inactivating the large 60S ribosomal subunits, thereby inhibiting protein synthesis. RIPs exert their toxic effects by depurinating a specific adenine base located in a universally conserved GAGA-tetraloop, present in rRNA [1, 2]. This results in the inability of the ribosome to bind elongation factor 2, thus irreversibly blocking protein translation. Plant RIPs have been widely employed to prepare immunotoxins (ITs), where the target selectivity can be achieved by using monoclonal antibodies recognizing antigens selectively expressed or over-expressed on the surface of target malignant cells. More recently, ligand targeted (LT) toxins have also been developed, where a specific targeting ligand (usually a growth factor domain) replaces the antibody domain [3, 4]. Among plant RIPs, saporin (SAP) is characterized by a high level of enzymatic activity, stability and resistance to conjugation procedures and blood proteases, that potentially make this toxin a very useful tool in cancer therapy. SAP-based ITs or LT fusions have been employed in preclinical studies with encouraging results and a few of them are under investigation in clinical trials [5]. Among the potential targets, a special place is deserved to the human urokinase-type plasminogen activator (uPA) and its receptor (uPAR) that have been demonstrated to be involved in the neoplastic evolution, including tumor cell proliferation, adhesion and migration, intravasation and extravasation, growth at the metastatic sites and tumor neo-angiogenesis. In particular, tumor tissue over-expression of uPAR has been shown to be associated with neoplasm aggressiveness and poor clinical outcome in several malignancies [6], identifying it as a good candidate target molecule for anti-cancer therapy. The first uPAR targeting peptide was created by isolating the N-terminal fragment (ATF) of uPA, containing the first 135 amino acids including the growth factor domain [7] that has been utilized as a target for therapy and imaging [8]. We have developed a chimeric protein consisting of the ATF of human uPA fused to saporin and shown it to specifically kill uPAR over-expressing malignant cells [9–11].

Several problems have been encountered in the production of ATF-SAP in bacteria and in *Xenopus laevis* oocytes due to limited peptide folding capabilities of prokaryotic cells together with overall host toxicities [9, 10, 12]. The yeast expression system represents a significant step forward in the production of the ATF-SAP chimera [12]. Yeast cell factories combine the advantages of being single cells, combining rapid growth with easy genetic manipulation, together with important characteristic features of eukaryotic cells that include a secretory pathway that leads to protein processing and post-translational modifications [13]. In particular, the methylotrophic yeast *Pichia pastoris* has been utilized for the production of hundreds heterologous polypeptides since it recapitulates most of the co- and post-translational events during protein translation; indeed, the presence of the endoplasmic reticulum quality control system allows only secretion-competent polypeptides to reach the extracellular medium and assures a proper oxidative folding process [14]. Moreover, straightforward purification of secreted recombinant proteins is possible due to the relatively low levels of native secreted proteins. Another relevant issue is that this yeast is a non-pathogenic organism and recombinant protein production is effectively pyrogen-free (unlike *E. coli* derived proteins), making it safer for human use. The fermentation process can be readily scaled up to meet greater demands, and parameters that influence protein productivity and activity, such as pH, aeration and carbon source feed rate, can be readily controlled [13]. The yield of secreted protein can be increased by utilizing a multistage process where initially the biomass is expanded, and secondly the expression of the gene of interest is induced, resulting in high titer and yield of bioconversion by a viable but resting biomass of cells [14]. This strategy is particularly effective when the product of interest is toxic towards the recombinant hosts, particularly when using GS115 (his4) strain [2].

In the present work, the overall optimized codon sequence for the saporin-based chimera ATF-SAP was expressed in *P. pastoris* under the control of the methanol inducible AOX1 promoter. We performed the optimization of cell growth and protein expression using shake flask cultures. Afterwards, we performed the production in a stirred tank bioreactor using methanol additions as a feeding strategy, which led to our obtaining increased levels of recombinant chimeric fusion in the supernatants. The ATF-SAP protein purified from the fermentation supernatants was specifically cytotoxic in vitro against a tumor cell line model over-expressing the target receptor uPAR.

## Results

### Molecular design, expression, and optimization of the chimeric fusion ATF-SAP

Due to host toxicity, misfolding and degradation of the ATF domain during ATF-SAP expression in bacteria it was not practically possible to develop this chimeric fusion in this expression system. To overcome these limitations, recombinant ATF-SAP has been produced in the methylotrophic yeast *P. pastoris* [12], allowing the correct formation of disulphide bonds in the ATF moiety and possessing little or no toxicity due to efficient segregation of the fusion protein to the endoplasmic reticulum and subsequent secretion in the culture medium.

**Fig. 1:**
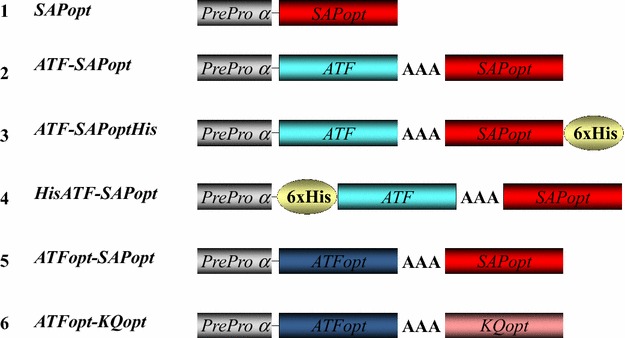
Constructs for the expression of ATF-SAP in *Pichia pastoris* yeast. Schematic representation of SAP (*1*) or ATF-SAPs (*2*–*6*) chimeric fusions. Native ATF (*light blue*), or optimized for the expression in yeast (ATFopt, *blue*) were fused through a 3 alanine linker to optimized SAP WT (*red*) or mutant SAP KQ (*pink*). A hexahistidine tag was appended either at the C- (construct 3) or at N-terminus (construct 4)

With the objective of scaling up the production of the saporin-based therapeutic molecule ATF-SAP in bio-fermenters, we prepared and compared a panel of ATF-SAP constructs (Fig. 1) either carrying an N- or C-terminal hexahistidine tag.

**Fig. 2:**
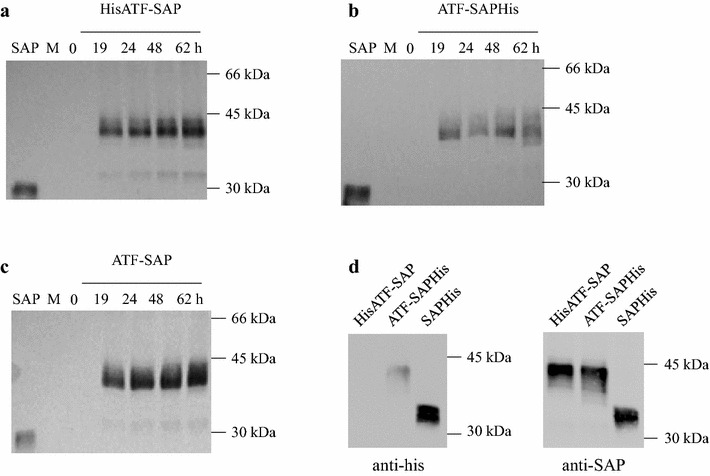
Time course analysis of the expression of ATF-SAP fusions in *Pichia pastoris*. Best-expresser clones for HisATF-SAP (**a**), ATF-SAPHis (**b**) and ATF-SAP (**c**) were induced and protein production assessed at different time points by western blotting using anti-saporin antibody as a probe. Equal amounts of culture supernatants were loaded. Seed derived SAP was used as a control. The presence of the histidine tag in ATF-SAPs was confirmed by western blotting using an anti-histidine antibody (**d**, *left panel*) and anti-SAP antibody as control (**d**, *right panel*)

We have previously established that the GS115 yeast strain can better tolerate the production of active saporin polypeptides, showing essentially no major differences between the expression levels of SAP WT after codon optimization and its catalytically inactive mutant SAP KQ, generated by mutation of two key active site residues (E176 and R179 in mature SAP) [15]. Indeed, the codon usage optimization markedly increased the number of clones that were characterized by a high level of SAP expression [12]. Moreover, it has been demonstrated previously that the human ATF can be efficiently folded and secreted by *P. pastoris* [16]. We therefore, took advantage of this for expressing optimized ATF-SAP, containing the saporin sequence optimized for yeast expression [12]. To assist the protein purification process, we prepared two different histidine-tagged constructs harbouring an hexahistidines tag at the N-terminus (HisATF-SAP) or at the C-terminus (ATF-SAPHis) domain (Fig. 1). One objective was to compare these his-tagged constructs to an ATF-SAP, without the tag, and thus to determine whether the presence of a hexahistidine tag might influence the production level, intracellular transport and catalytic activity of the chimeric toxin. These constructs also contained the Kex2-mediated endoproteolytic cleavage site (Lys-Arg) required for proper processing of the prepro-alpha N-terminal domain of the fusion protein and an (Ala)_3_-linker between the ATF domain and saporin. DNA constructs were cloned into the integrative pPICZalpha plasmid for heterologous expression under the control of the methanol inducible alcohol oxidase promoter (AOX). The advantage of these vectors, despite their low copy numbers, is the robust genetic stability of the resulting transformants, even in unselective medium. For the selection of the best ATF-SAP expressing clones, supernatants from different yeast cultures expressing HisATF-SAP, ATF-SAP or ATF-SAPHis after 24 h of induction were transferred to nitrocellulose filter and incubated with anti-SAP antibody. In light of the results obtained (Additional file 1: Fig. S1), we chose the clone that showed the highest expression for each chimera (clones 2, 34 and 29 for HisATF-SAP, ATF-SAPHis and ATF-SAP, respectively) in order to perform a small-scale fermentation. Accumulation of recombinant proteins was monitored in time course experiments that showed a substantial accumulation in the medium during the first 24–48 h post-induction (Fig. 2a–c). We then analyzed the protein products to verify the presence of the His-tag, but little or no signal in the HisATF-SAP and ATF-SAPHis was revealed in the samples decorated with anti-His antibody (Fig. 2d), indicating that that the chimera containing a histidine tag at the N-terminus might be processed by exo-or endo-peptidases. Reactivity with the anti-his antibody appeared to be reduced, although not abolished, also in the case of ATF-SAPHis (Fig. 2d). Since the his-tag appeared to be partially or totally lost, we chose the untagged ATF-SAP fusion for further development.

**Fig. 3:**
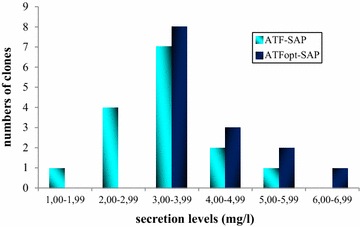
Analysis of ATF-SAP expression in *Pichia pastoris* after codon usage optimization of the ATF domain. Histograms showing the distribution of 14 (ATFopt-SAP, *dark blue*) and 15 (ATF-SAP, *light blue*) independent clones induced for 24 h for expression of native or optimized (opt) ATF-SAP. Expression levels are reported in different ranges from the lowest (1.00–1.99 mg/l) to the highest expressers (6.00–6.99 mg/l)

We have previously demonstrated the importance of yeast codon-usage optimization to increase the number of highly expressing saporin clones on the background of GS115(his4) yeast cells [12], by avoiding potential depletion in tRNAs for highly frequent amino acids and possibly increasing the rate of recombinant protein synthesis. Accordingly, we designed, amplified and cloned a synthetic gene encoding ATF sequence optimized for yeast expression (ATFopt), in order to obtain, in this way, both the components of the chimera fully optimized. Several independent clones derived from transformed *P. pastoris* GS115 yeast were induced and ATF-SAP secretion was monitored (Fig. 3). As in the case of SAP [12], the optimization of the sequence encoding the ATF domain prompted a shift in the distribution of the clones towards groups of higher expressors. The same strategy was employed to generate the catalytically inactive ATF-SAP KQ fully optimized.

### Production of recombinant ATF-SAP chimeras

**Fig. 4:**
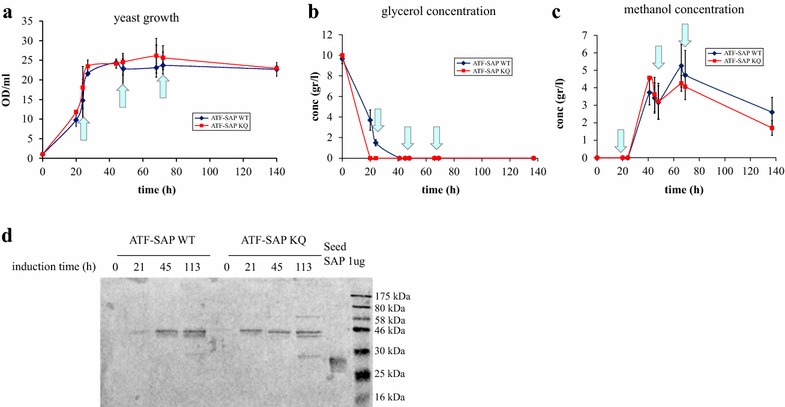
Shake flask production of ATF-SAP WT and ATF-SAP KQ. *Pichia pastoris* growth curve (**a**), glycerol (**b**) and methanol (**c**) concentration were monitored before and after the induction of ATF-SAP WT (*blue line*) or ATF-SAP KQ (*red line*) expression. The *light blue arrows* indicate the points at which pulses of methanol were delivered. *Error bars* represent standard deviations from the mean of triplicate samples. **d** The chimeric fusions in the supernatants at the indicated time points following induction were analyzed by western blotting using anti-saporin antibody as probe

A production protocol for the best construct obtained was performed by growing independent transformants of *P. pastoris* GS115 expressing ATF-SAP WT or its inactive mutant ATF-SAP KQ. In the batch phase the initial concentration of glycerol was 10 g/l in order to obtain a significant level of biomass prior to triggering the expression of the recombinant gene. It is known that the maximum specific growth rate of *P. pastoris* on methanol is, generally, lower when the yeast is producing a heterologous protein because of the negative effect that the metabolic burden of heterologous protein production exerts on the cells [17], as it proved to be in our case (data not shown). The production of chimeras was triggered by the daily addition of methanol and growth was monitored for 4 days. The growth curves were very similar between the two strains, indicating that the ATF-SAP WT produced and accumulated in the culture medium had little or no toxicity for the yeast, even after 92 h of induction (Fig. 4a). The titers of substrates were monitored over time by HPLC (Fig. 4b, c). Of note was that, all (in the case of ATF-SAP KQ) or most (ATF-SAP WT) glycerol was depleted before the ATF-SAP production was induced by the addition of methanol. The production of both ATF-SAP WT and ATF-SAP KQ chimeras increased by time and the titer was estimated around 10–11 mg/l (Fig. 4d). A small proportion of both secreted proteins was degraded at the latest time point, as a band co-migrating with a control seed saporin around 30 kDa.

### Production of recombinant ATF-SAPs in bioreactor

**Fig. 5:**
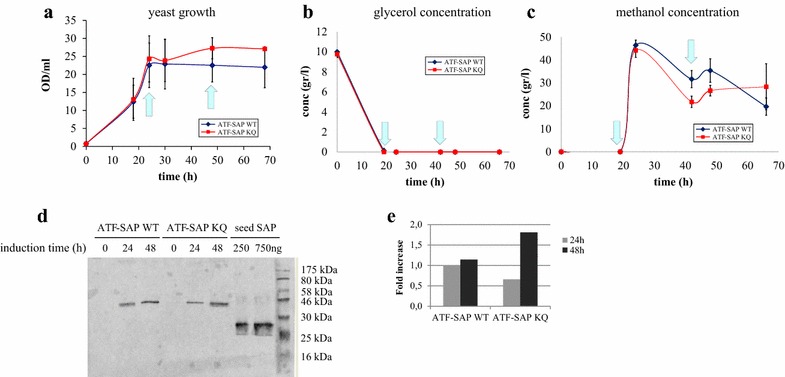
Production of ATF-SAPs in bioreactor. *Pichia pastoris* growth curve (**a**), glycerol (**b**) and methanol (**c**) concentration were monitored before and after the induction of ATF-SAP WT (*blue line*) or ATF-SAP KQ (*red line*) expression. The* light blue arrows* indicate the pulses of methanol. *Error bars* represent standard deviations from the mean of triplicate samples. The chimeric fusion proteins secreted into the supernatants at the indicated time points were analyzed by western blotting using an anti-saporin antibody as probe (**d**). Bands were quantified and expressed as fold increase (**e**)

We then proceeded to further develop the bioprocess in order to both check the performance of the optimized ATF-SAP and evaluate the possibility to produce the chimera in bioreactors. *P. pastoris* GS115 transformants expressing ATF-SAP WT or ATF-SAP KQ were grown in 2 l stirred tank bioreactors. The fermenters were equipped with oxygen and pH electrodes to monitor and control oxygen consumption and pH by adjusting stirring and KOH addition, respectively. Besides these, other parameters influencing protein productivity and activity, such as temperature and carbon source feed rate, were monitored. Yeasts were cultured in BMGY medium supplemented with histidine, at constant temperature of 30 °C and pH of 5.5. It has been previously shown that, during the yeast growth, the medium quickly acidifies and the acidification inhibits cell growth and metabolism, even if low pH values increase the production of recombinant proteins through a reduction in protease activity in the cultures [18]. To minimize medium acidification and optimize cellular growth and protein production, we buffered the cultures to pH 5.5. To support the oxygenation of yeast cells and thus their catabolism, stirring was set up to be proportionally increased in case of pO_2_ reduction under 25%. The carbon availability was indirectly reflected by changes in the concentration of dissolved oxygen. To mimic the production strategy previously described, the first 24 h were dedicated to increase the biomass with cells growing and entirely consuming glycerol, the carbon and energy source (Fig. 5). After testing different concentrations of glycerol in the fermentation medium, from 10 to 40 g/l, we chose 10 g/l since cells could consume this amount of substrate in about 20–24 h. Glycerol depletion is a critical factor since we could not induce any ATF-SAP production when the methanol addition preceded its consumption. Protein production was induced by pulse addition of 0.5% methanol every 24 h. Since growth on methanol is expected to generate heat and increase oxygen consumption, temperature and oxygen levels were monitored by the system and spikes that occurred were rapidly adjusted. The western blotting analysis of culture media revealed the presence of distinct bands corresponding to the chimeric fusion proteins accumulating over time (Fig. 5d), confirming the efficient induction of the recombinant constructs following methanol supplementation. In addition, this analysis confirmed also that during production in the bioreactor the recombinant proteins did not undergo detectable degradation (Fig. 5d). The concentration of the expressed recombinant proteins calculated by densitometric analysis, was approximately 6 mg/l for ATF-SAP WT and 7 mg/l for ATF-SAP KQ after 48 h of induction, slightly less than compared to those obtained in flasks.

**Fig. 6:**
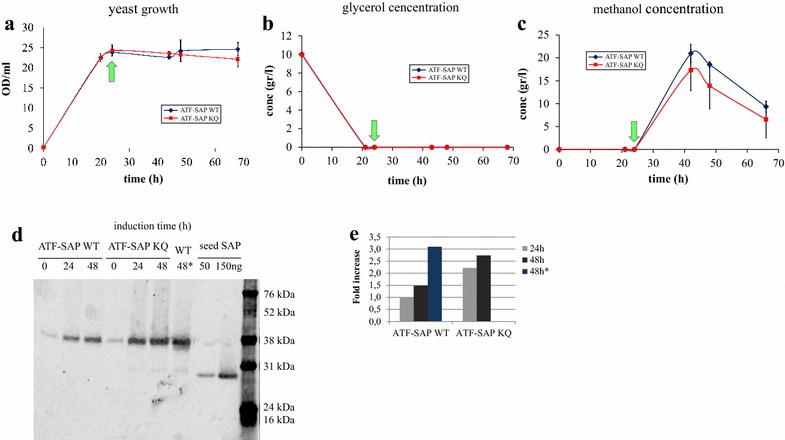
Fed-batch production of ATF-SAPs. *Pichia pastoris* growth curve (**a**), glycerol (**b**) and methanol (**c**) concentration were monitored before and after the induction of ATF-SAP WT (*blue line*) or ATF-SAP KQ (*red line*) expression. The* green arrows* indicate the methanol fed-batch. *Error bars* represent standard deviations from the mean of triplicate samples. The chimeric fusions secreted in the supernatants at the indicated time points were analyzed by western blot with anti-saporin antibody (**d**). Bands were quantified and expressed as fold increase (**e**). The *asterisk* indicates a different fed-batch production

To increase the productivity and reduce the possible toxicity due to a methanol pulse, a different approach based on fed-batch addition of methanol was conducted. Indeed, a feeding strategy could allow the cells to adapt smoothly on methanol. After glycerol consumption, the fed-batch phase of methanol was achieved using a constant flow rate of 7.5 ml/h for 24 h. In these conditions, the curves of growth and glycerol concentration of both ATF-SAP WT and ATF-SAP KQ were almost completely superimposable (Fig. 6). The production of the inactive mutant construct was greater than the wild type (Fig. 6d): the titer of recombinant protein production was approximately 6 mg/l for ATF-SAP WT and 11 mg/l for ATF-SAP KQ after 48 h. To improve the titer of ATF-SAP WT, a further optimization was performed by increasing the oxygen transfer rate by enhanced stirring (data not shown), since in the production strain the oxygen requirement upon methanol addition is higher than in the control strain. Glycerol concentration and growth were comparable with previously conducted experiments (data not shown), but the protein production was significantly improved to 12.5 mg/l (Fig. 6d, line 7, the band marked with an asterisk). Glycerol consumption, growth rate and correlation between OD and cell dry weight are shown in Table 1.

**Table 1:** Growth and ATF-SAP production parameters in *P. pastoris* recombinant cells during fed-batch fermentation

	Growth rate (h^−1^)	Glycerol uptake rate (g/l/h)	Correlation OD_660nm_/cell dry weight^a^	ATF-SAP yield (mg/g)^b^
ATF-SAP WT	0.42 ± 0.01	0.52 ± 0.01	y = 0.1853x^2^ + 0.3104x	0.98 ± 0.03
ATF-SAP KQ	0.44 ± 0.02	0.54 ± 0.02	y = 0.1956x^2^ + 0.4885x	0.86 ± 0.04

^a^y = OD_660nm_; x = cell dry weight, mg

^b^calculated on yeast dry weight at 48 h after induction

As shown by the kinetic profiles, the strains harboring the ATF-SAP KQ or ATF-SAP WT construct do not differ significantly, demonstrating that the developed cell factory can efficiently support the production of the catalytically inactive as well as of the active construct. Minor differences can be recorded in the oxygen and stirring profiles (Additional file 2: fig. S2C and S2D), indicating a slightly better performance of the strain harboring the ATF-SAP KQ construct, which nevertheless does not result in significant differences in terms of recombinant protein production (titer). The overall data demonstrate that the ATF-SAPs fed-batch production in bioreactors was successfully achieved, and that improvements in protein titer can be obtained in this way, opening the possibility to produce discrete homogenous batches for in vitro experiments on cells.

### Purification and cytotoxic activity of recombinant ATF-SAPs

We then investigated if the chimeric fusion proteins produced in the bioreactors were fully active for potential use in the future as anti-cancer therapeutics.

**Fig. 7:**
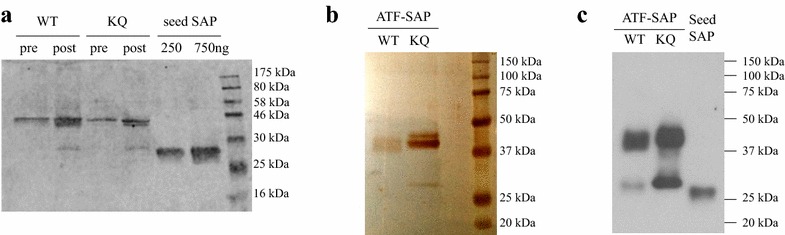
Characterization of ATF-SAP produced in *P. pastoris* and purified by IMAC. Yeast supernatants containing ATF-SAP WT and ATF-SAP KQ (pre) were treated with ammonium sulphate for protein precipitation and dialyzed (post). Protein products were analyzed by western blotting with an anti-saporin antibody (**a**). ATF-SAPs purified by IMAC were detected by silver staining (**b**) and western blotting (**c**) using an anti-saporin antibody as probe

Simple purification of secreted recombinant proteins is possible due to the relatively low levels of native secreted proteins in *P. pastoris* [19]. Supernatants from fed-batch fermentations of ATF-SAP WT and ATF-SAP KQ expressing cells were treated with a saturated solution of ammonium sulphate, to precipitate proteins. Being highly soluble and a strong salting-out agent, ammonium sulphate is among the most successful additives for protein crystallization [20]. Pellets derived from salting-out were resuspended in PBS and dialyzed. The presence of concentrated ATF-SAPs was assayed and, as shown in Fig. 7a, both ATF-SAP WT and ATF-SAP KQ were both efficiently precipitated out from the fermentation supernatants. To purify saporin-based chimeras, samples were then loaded on a cation ion-exchange chromatography column and eluted with a linear NaCl gradient. The obtained chimeric proteins were analyzed following silver-staining of SDS-PAGE gels and by western blotting using an anti-saporin antibody as probe for accurate protein quantifications (Fig. 7b, c). ATF-SAP was by far the most abundant product representing more than 95% of the total purified protein (Fig. 7b). In addition to the intact ATF-SAP fusion protein with the expected molecular weight of 44 kDa, another faint band at 30 kDa, most likely corresponding to saporin, was also detected (Fig. 7c). A summary of purification efficiency is reported in the Additional file 3: Table S1.

**Fig. 8:**
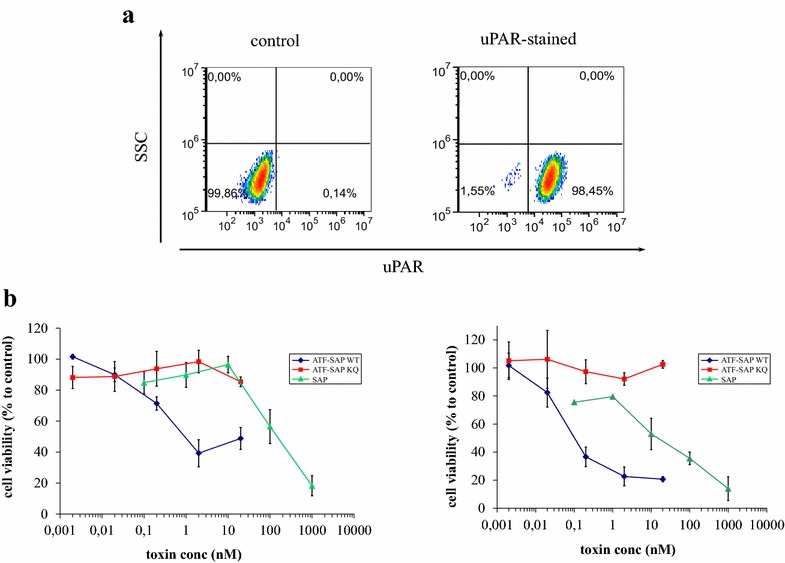
Cytotoxic activity of ATF-SAP was assayed on a uPAR over-expressing cancer cell line. Flow cytometric analysis of the cell surface expression of uPAR in the human leukemia U937 cell line using an anti-uPAR antibody (**a**). The secondary antibody used alone acted as control. **b** U937 cells were exposed to increasing concentrations of ATF-SAP WT (*blue*), ATF-SAP KQ (*red*) or seed saporin (*green*) and cell viability was measured by MTT assay after 48 (*left panel*) or 72 h (*right panel*). *Error bars* represent standard deviations from the mean of triplicate samples

To test the activity of purified ATF-SAP WT and ATF-SAP KQ, we performed a preliminary experiment on LB6 clone19 mouse fibroblast cells stably expressing human uPAR and previously shown to be sensitive to ATF-SAP [9]. LB6 cells were exposed to increasing concentrations of purified ATF-SAP chimeric proteins or to seed SAP as a control and their cytotoxicity was compared in a MTT assay. ATF-SAP WT was one hundred times more cytotoxic than unconjugated seed SAP alone and the catalytically inactive mutant ATF-SAP KQ did not exert any cytotoxicity over the concentration range studied (Additional file 4: Fig. S3). To validate our system on human cancer cells, we tested the activity of ATF-SAP on the U937 leukaemia cell line. U937 over-expresses uPAR, as shown in Fig. 8a, and represents an appropriately relevant target for ATF-SAP. uPAR-directed saporin chimera has been demonstrated to be highly active on U937 [9–11]. U937 cells that were treated at different time points with increasing concentrations of toxins and ATF-SAP WT demonstrated an IC_50_ of 0.95 nM at 48 h that further decreased a further ten-fold after 72 h of treatment (0.1 nM). Seed saporin was observed to be markedly less toxic (IC_50_ around 150 nM at 48 h and 15 nM at 72 h) than ATF-SAP WT and the inactive mutant ATF-SAP KQ did not exhibit any cytotoxicity over the concentration range studied (Fig. 8b). Our overall results demonstrate that the production of uPAR-targeted, saporin-based recombinant fusion ATF-SAP that has significant therapeutic potential in the treatment of human cancers can be efficiently produced into a 2 l bioreactor and that the obtained chimeric protein is has full cytotoxic activity against human cancer cells.

## Discussion

Recombinant protein production is a multibillion-dollar market, comprising biopharmaceuticals and industrial enzymes. A crucial step in the development of a new product is the choice of the production host. While one single perfect host for every protein does not exist, several expression systems ranging from bacterial hosts to mammalian cells have been established. Since 1992, when insulin, the first recombinant product of DNA technology was launched on the world market, many proteins such as interferons, erythropoietin, vaccines, and more recently monoclonal antibodies and industrial enzymes have followed. In the subsequent years, many efforts have been made to improve the production yield and to find new organisms capable of allowing proper expression of active polypeptides. This issue is especially relevant for toxin-based therapeutics, considering that they are poisonous for the expression host. Among the various expression systems, yeast cells have emerged as a substantial factory for biotechnological drugs, owing to their special features including rapid growth, simple molecular manipulations, together with their ability to secrete heterologous proteins that undergo ER quality control for correct folding, processing and post-translational modifications [13]. We have formerly shown that ATF-SAP, a chimeric fusion formed between the toxin saporin and the amino-terminal binding domain of urokinase can be produced in *P. pastoris* yeast, without any major toxicity within the host cells [12]. We have now further optimized the ATF-SAP construct for expression in yeast in a fermentation process using lab-scale stirred bioreactor to produce discrete batches of recombinant ATF-SAP that, following purification, was demonstrated actively cytotoxic for uPAR over-expressing U937 cells. Notably, the codon usage optimization of ATF domain determines an increased number of ATF-SAP highly expressing clones by the GS115 yeast strain, an observation in line with the improvement obtained with the optimization of SAP encoding sequence [12]. The GS115 yeast strain expressed ATF-SAP WT, and the catalytically inactive mutant ATF-SAP KQ (containing KQ saporin), even in the fed-batch fermentation, demonstrating the tolerance of this strain towards SAP, as previously reported [12]. In contrast, examples of toxicity of diphtheria toxin-based fusions expressed in *P. pastoris* have been reported both in terms of marked reduction in the growth rate and significant loss of viable cells [21, 22]. In the present study, we did not detect any intracellular ATF-SAP in the yeast cells showing that leakage from the secretory pathway did not occur (data not shown). This observation was corroborated by the fact that the growth curves of ATF-SAP WT and the inactive mutant KQ were superimposable in the fed-batch fermentations.

The titer of a recombinant protein produced in *P. pastoris* is a function of several factors such as pH, temperature, dissolved oxygen, growth rate and induction medium. As there is no single set of culture conditions that will work optimally for any type of recombinant protein, we assessed different parameters in small-scale production processes to translate our findings towards further optimization (for use) in stirred tank bioreactors. In the fermentation process utilized in this *Pichia* expression system, all the glycerol needs to be consumed before starting the methanol feed to fully derepress the AOX1 promoter to induce recombinant protein production. Methanol feeding proved to be a better way to induce production of ATF-SAPs in general compared with pulse addition; in this instance yeast cells better adapted to methanol and the titer of protein was higher when starting from comparable biomasses. The maximal production of recombinant ATF-SAP was obtained after 48 h of induction; additional time did not increase the titer. However, degradation products appearing in the fermentation medium did increase further with time, likely due to both the total methanol consumption and the activity of membrane-bound or secreted proteases. We cannot exclude that the titer obtained could be further ameliorated. However, a limit to production levels of recombinant protein is likely determined by the concentration of methanol used for induction that above a threshold value proves to be too toxic for the yeast. We believe that further improvements would be possible by boosting the pre-induction biomass formation, such as achievable in industrial-scale bioreactors. The *P. pastoris* expression system has gained importance for industrial application as highlighted by the number of patents published on heterologous protein expression [23], some of which have already found their way to the market, as FDA approved biopharmaceuticals or industrial enzymes [24].

We have demonstrated that the cytotoxicity of the chimeric ATF-SAP is unambiguously due to the RIP activity of saporin, as the inactive mutant ATF-SAP KQ failed to exert any cytotoxic effect on U937 target cells. ATF efficiently delivers saporin to uPAR-overexpressing U937 cancer cells, because ATF-SAP has an IC_50_ one hundred-fold lower than saporin, confirming the targeting efficiency of this ligand. We do not anticipate that the presence of proteolytic-derived material comprising less than 5% of the total purified chimeric protein may affect the results of the cytotoxicity experiment in U937 in terms of a significant reduction of the ATF-SAP toxicity.

The potential therapeutic application of ATF-SAP is huge, ranging from hematological tumors to solid cancers that frequently show an increase of uPAR expression, in particular when tumors became more aggressive, invade the surrounding tissues and start the metastatization process [25, 26]. Indeed, uPAR has been proposed as a diagnostic marker in clinical practice [8] and has already been employed to target bacterial protein toxins to glioblastoma multiforme [27]. SAP is characterized by high enzymatic activity, stability and resistance to proteolytic degradation; it is less immunogenic compared to bacterial toxins and well tolerated [28]. On account of these key features SAP has been widely used to produce very efficient conjugates and chimeric fusions for the killing of target cells currently undergoing preclinical or clinical evaluation, mostly in the context of neoplastic diseases.

This study demonstrates that *P. pastoris* is a suitable expression system for the bioreactor based fed-batch fermentation process to efficiently produce ATF-SAP under methanol-inducible AOX1 promoter and paves the way for the industrial development of the production process for this chimeric fusion protein, a potentially powerful therapeutic molecule for cancer therapy.

## Methods

### Preparation of DNA constructs

All constructs are based on the pPICZalpha vector (Invitrogen) used for the heterologous protein expression in yeast. pSAP and pATF-SAPHis with the sequence encoding saporin optimized for the expression in yeast have been previously generated [12]. pATF-SAP plasmid was prepared by *Xho*I/*Not*I double digestion of pATF-SAPHis to recover the ATF sequence to insert in the *Xho*I/*Not*I cut pHis4KBoptG4S-SAPopt [29] recipient vector. The pHisATF-SAP was obtained by the insertion of an ATF fragment generated by PCR (PmlIATF forward primer 5AGCAATGAACTTCATCAAGTTCCATCGAAC; ATFRevNotI reverse primer CAGTAGCGGCCGCTTTTCCATCTGC) into the pHis4KBoptG4S-SAPopt plasmid digested with *Pml*I and *Not*I. To generate the pATFopt-SAP, the codon-optimized DNA sequence encoding ATF was custom synthesized by Genscript Corporation (Piscataway, NJ, USA) and inserted in the *Xho*I-*Not*I cut pHis4KBoptG4S-SAPopt. The pATFopt-SAP KQ was generated by introducing the ATFopt sequence in the *Xho*I-*Not*I-cut pITKQ recipient vector followed by ATF-SAP KQ subcloning to pPicZalphaA vector by *Xho*I-*Xba*I digestion.

### Transformation, selection, and induction of *P. pastoris* clones

Electrocompetent *P. pastoris* GS115 (his4) cells were prepared according to Invitrogen protocols. A Bio-Rad Gene pulser apparatus (Bio-Rad, Milan, Italy) was used for electroporation. The DNA constructs expressing HisATF-SAP, ATF-SAP or ATF-SAPHis were linearized for genomic integration before electroporation using *Hin*dIII restriction enzyme. Five micrograms of linearized plasmids were used for each electroporation cuvette. Linearized empty pPICZalpha vectors were used for the mock-transformed cells. Then, 500 µl of transformed cells was plated for selection on YPD [1% (w/v) yeast extract, 2% (w/v) peptone or tryptone, and 2% (w/v) dextrose] plates containing 18.2% sorbitol (YPDS) in the presence of 2% (w/v) agar and 75 µg/ml Zeocin (Invitrogen) for 3 days at 30 °C. Randomly selected colonies were restreaked onto YPDS-zeocin plates. For selecting the best-expressor clones, 40 colonies underwent a colony-lift procedure as described [12]. Preliminary production in shake flasks were performed as described [12]. Briefly, clones were grown in 5 ml YPD with 50 µg/ml Zeocin at 30 °C for 16 h. Cells were centrifuged and resuspended in 5 ml BMMY [1% (w/v) yeast extract; 2% (w/v) peptone; 100 mM phosphate buffer, pH 6.0; 1.34% (w/v) yeast nitrogen base, 4 × 10^−5^% (w/v) biotin; 4 × 10^−3^% (w/v) histidine and 0.5% (v/v) methanol] to a final OD600/ml of 2.0 in 50 ml conical tubes. Noninduced cells were resuspended in 5 ml of BMDY, which is the same as BMMY except that 2% (w/v) dextrose is present instead of methanol. Cultures were noninduced or induced for 48 h at 30 °C with shaking at 250 rpm, and further 0.5% (v/v) methanol was added after 24 h. Best expressors were grown 16 h in liquid culture and cells (10–15 OD600) were centrifuged, resuspended in 1 ml of YPD supplemented with 15% sterile glycerol, and frozen immediately. Stocks were stored at −80 °C until use. The best clones (one for ATF-SAP WT and one for ATF-SAP KQ) were then used for production in 2 l shake flasks. Briefly, glycerinate stocks have been grown in 20 ml YPD medium overnight at 30 °C, 250 rpm. Cells were then inoculated in 200 ml YPD at initial OD_660_ = 1 at 30 °C, 250 rpm. Exponentially growing cells have been inoculated in 400 ml BMGY medium [1% (w/v) yeast extract; 2% (w/v) peptone; 100 mM phosphate buffer, pH 6.0; 1.34% (w/v) yeast nitrogen base, 4 × 10^−5^% (w/v) biotin; 4 × 10^−3^% (w/v) histidine, 10 gr/l glycerol]. Three pulses of 2 ml pure methanol have been performed at 24, 48 and 69 h from the inoculum (final methanol concentration: 0.5% v/v). The ratio of flask volume:medium was 5:1. Samples (1 ml) were taken at different times post induction for protein expression analyses. At the end of the induction period, the medium was collected after centrifugation at 10,000*g*, 15 min at 4 °C and kept frozen at −80 °C before thawing at 30 °C for subsequent protein purifications.

### Bioreactor fermentation cultures

One hundred milliliters of pre-culture was grown overnight at 30 °C in YPD medium. The culture was centrifuged at 6000 rpm for 10 min and the harvested cells were resuspended in 1 l BMGY and used to inoculate a 2 l stirred tank bioreactor (Braun Biotech, Melsungen, Germany) at an initial optical density of 0.2. Bioreactors were equipped with in situ pH and polarographic dissolved oxygen electrodes, temperature sensors and stirring speed control. Cells were cultured under the following conditions: temperature was maintained at 30 °C, pH was automatically controlled by KOH 2 N at 5.5 and stirring speed was in cascade to 25% of dissolved oxygen, therefore increasing upon oxygen consumption. Two different induction strategies have been adopted. In one case the production was induced with two feeding of 50 ml pure methanol (pump rate set on 0.125 ml/min, 6 h 40 min for each addition) at 19 and 48 h from the inoculum. In the other case, after 24 h, when glycerol was exhausted, the methanol fed-batch phase was initiated (total added methanol volume: 100 ml). The pump rate was set on 0.125 ml/min. At the end of fermentation process yeast cultures were centrifuged at 10.000*g* for 30 min at 4 °C and supernatants were stored at −80 °C. Biomass analysis was performed by measuring the optical density of yeast cultures at a wavelength of 600 nm. Both pulse and fed-batch fermentations have been performed at least 3 times and variations are shown as average ± standard deviation.

The amount of extracellular methanol and glycerol was determined by High-Performance Liquid Chromatography (HPLC) based method using 5 mM H_2_SO_4_ as mobile phase and Aminex HPX-87P column, 300 × 7.8 mm with a polystyrene divinylbenzene-based matrix (BioRad). Concentrations were determined by comparison with pure standard samples.

### Protein purifications

*Pichia pastoris* culture media containing ATF-SAP WT or ATF-SAP KQ after methanol induction were centrifuged to remove cells. Supernatants were filter sterilized and proteins were precipitated by adding dropwise a cold, 100% saturated (3.9 M, pH 6.8) ammonium sulphate solution to reach a 80% final concentration. After 1 h incubation in ice with gentle stirring, samples were centrifuged at 10000*g* for 30 min at 4 °C. Pellets were resuspended in PBS and dialyzed over night at 4 °C in Membra-Cell MD 25-14 dialysis membrane (14,000 KDa cut-off) against PBS. Dialyzed material was further purified by ion exchange chromatography through CM Sepharose Fast Flow column (GE Healthcare) and eluted with PBS/1 M NaCl. Eluates were exchanged against PBS as above and filter sterilized.

### SDS-PAGE, Western, slot blot and silver-staining analysis

SDS-PAGE was performed on 12% polyacrylamide gels using the Laemmli buffer system. Proteins were silver stained or transferred onto nitrocellulose membranes (Millipore) for Western blot analysis and probed with rabbit anti-saporin anti-serum 1:2000 or anti-His polyclonal antibody (Antibodies-online GmbH) and then with an anti-rabbit IRDye 680 secondary antibody 1:15.000 or an HRP-conjugated secondary antibody 1:20,000 followed by detection with ECL (Millipore). For quantitative determination, band intensity was measured by ImageJ software on images from silver-stained gels or non-saturated exposures of the films.

A Bio-Dot SF Microfiltration Apparatus (Bio-Rad) with Protran nitrocellulose membranes was used for slot blots, essentially following manufacturer’s instructions. Seed SAP was loaded as a reference standard in replicates.

### Flow cytometry analysis

Aliquots of 10^6^ human promyelocytic leukaemia U937 cells were stained with anti-uPAR antibody (1 h, RT) provided by American Diagnostica and then with a secondary Alexa Fluor 488 antibody. The secondary antibody only was used as negative control. Flow cytometric analysis was performed using Accuri C6 Flow cytometer (Becton–Dickinson) and data were analyzed by FCS Express.

### Cell culture and cell viability assay

U937 cells were maintained in suspension culture in RPMI 1640 medium supplemented with 10% (v/v) fetal bovine serum, 50 U/ml penicillin and 50 μg/ml streptomycin at 37 °C in a 5% CO_2_ humidified atmosphere.

U937 cells were plated in 96-well plates at a cell density of 2 × 10^4^ cells/well and treated with serial logarithmic dilutions of the purified ATF-SAP WT or ATF-SAP KQ ranking from 20 nM to 2 pM or seed saporin (from 1 µM to 0.1 nM) for 48 or 72 h. At the end of the exposure period, MTT assay was performed according to the manufacturer’s recommendations (Sigma-Aldrich). Purple formazan product was resuspended in DMSO and measured in a spectrophotometric microplate reader at 570 nm wavelength. Each experiment was performed in quadruplicate and data were reported as mean ± standard deviation. Cell viability was evaluated as the concentration inhibiting the 50% of growth with respect to untreated control cells and is expressed as IC_50_.

## Additional files

**Additional file 1: Fig. S1.** Analysis of the expression of ATF-SAP fusions in *Pichia pastoris.* Supernatants from yeast cultures expressing HisATF-SAP (A—light blue, clones 1–12), ATF-SAP (B—orange, clones 25–31) or ATF-SAPHis (B—red, clones 32–38) after 24 h induction were transferred to nitrocellulose filter and decorated with anti-SAP antibody. Known amounts of seed SAP (green) were used as standards. Mock treated (white) and non- induced samples (yellow, pink and brown) acted as negative controls

**Additional file 2: Fig. S2.** Record of oxygen percentage and stirring speed during protein production. Cultivation of *Pichia pastoris* producing ATF-SAP WT (A and C) or ATF-SAP KQ (B and D) in tank bioreactor upon pulse (A and B) or fed-batch (C and D) administration of methanol. Oxygen percentage are in blue and stirring speed in green

**Additional file 3: Table S1.** Efficiency of purification. Samples produced by yeast were quantified by western blotting. The total amount of the protein present in each step is indicated. The yield is expressed as the percentage of the protein with respect to the quantity produced by yeast

**Additional file 4: Fig. S3.** Cytotoxic activity of ATF-SAP was assayed on uPAR over-expressing cells. Murine fibroblasts (LB6) stably expressing human uPAR were exposed to increasing concentrations of ATF-SAP WT (blue), ATF-SAP KQ (red) of seed saporin (green) and cell viability measured by MTT assay after 72 h of treatment. Error bars represent standard deviations from the mean of triplicate samples

## Abbreviations

uPA: urokinase-type plasminogen activator
ATF: amino terminal fragments of uPA
uPAR: urokinase-type plasminogen activator receptor
SAP: saporin
RIPs: ribosome-inactivating proteins
IT: immunotoxin
AOX: alcohol oxidase
YPD: yeast extract peptone dextrose
BMGY: buffered glycerol-complex
