# Supplementary figures and images for: Optimization of construct design and fermentation strategy for the production of bioactive ATF-SAP, a saporin based anti-tumoral uPAR-targeted chimera
Source: Microb Cell Fact. 2016 Nov 14;15:194. doi: 10.1186/s12934-016-0589-1 (PMC5109808; doi:10.1186/s12934-016-0589-1)

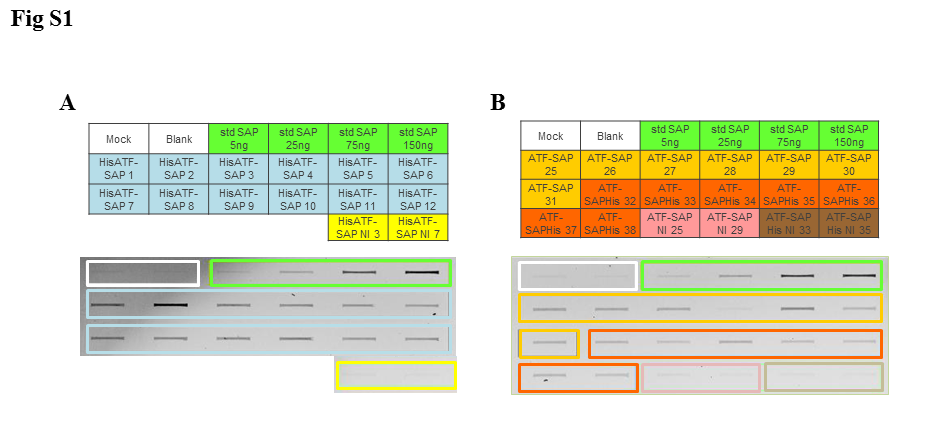

Supplement: Supplementary file 1 — Additional file 1: Fig. S1. Analysis of the expression of ATF-SAP fusions in Pichia pastoris. Supernatants from yeast cultures expressing HisATF-SAP (A—light blue, clones 1–12), ATF-SAP (B—orange, clones 25–31) or ATF-SAPHis (B—red, clones 32–38) after 24 h induction were transferred to nitrocellulose filter and decorated with anti-SAP antibody. Known amounts of seed SAP (green) were used as standards. Mock treated (white) and non- induced samples (yellow, pink and brown) acted as negative controls [file 12934_2016_589_MOESM1_ESM.tif]

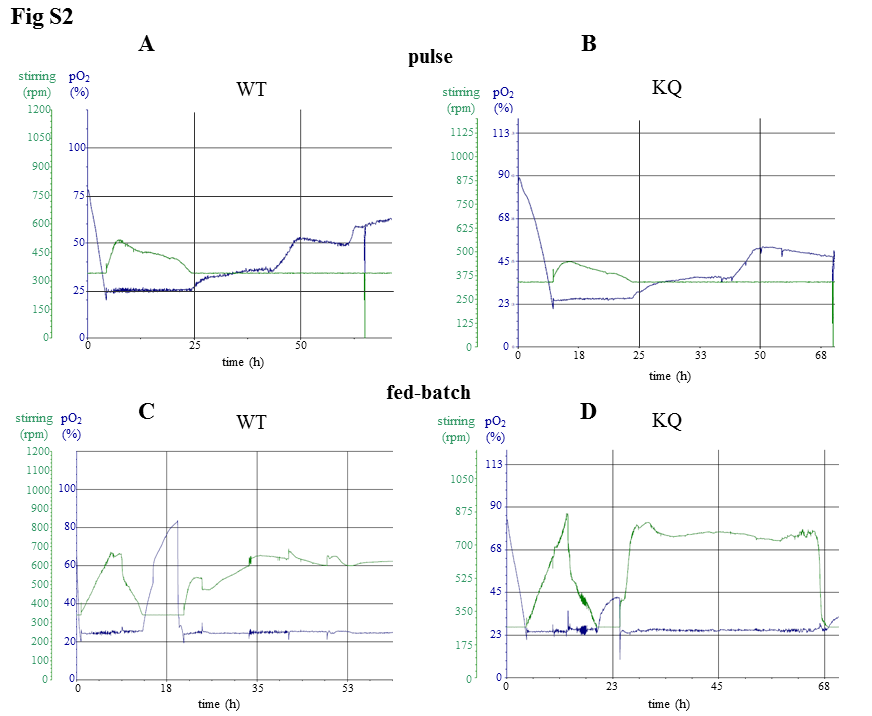

Supplement: Supplementary file 2 — Additional file 2: Fig. S2. Record of oxygen percentage and stirring speed during protein production. Cultivation of Pichia pastoris producing ATF-SAP WT (A and C) or ATF-SAP KQ (B and D) in tank bioreactor upon pulse (A and B) or fed-batch (C and D) administration of methanol. Oxygen percentage are in blue and stirring speed in green [file 12934_2016_589_MOESM2_ESM.tif]

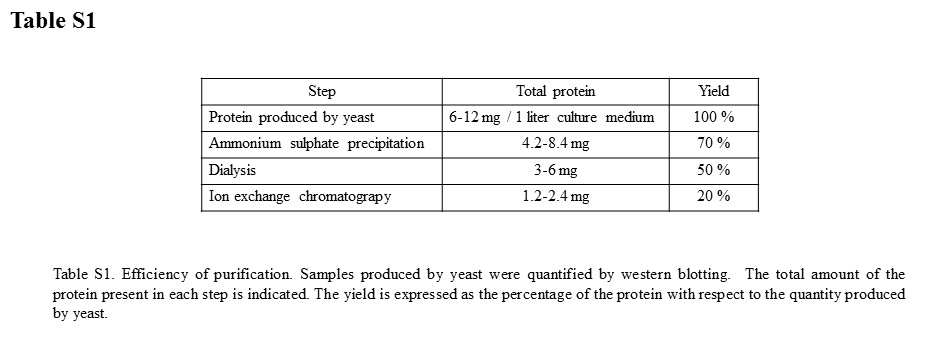

Supplement: Supplementary file 3 — Additional file 3: Table S1. Efficiency of purification. Samples produced by yeast were quantified by western blotting. The total amount of the protein present in each step is indicated. The yield is expressed as the percentage of the protein with respect to the quantity produced by yeast [file 12934_2016_589_MOESM3_ESM.tif]

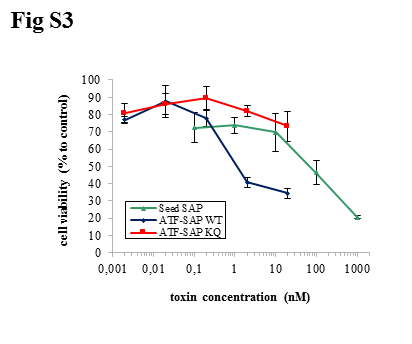

Supplement: Supplementary file 4 — Additional file 4: Fig. S3. Cytotoxic activity of ATF-SAP was assayed on uPAR over-expressing cells. Murine fibroblasts (LB6) stably expressing human uPAR were exposed to increasing concentrations of ATF-SAP WT (blue), ATF-SAP KQ (red) of seed saporin (green) and cell viability measured by MTT assay after 72 h of treatment. Error bars represent standard deviations from the mean of triplicate samples [file 12934_2016_589_MOESM4_ESM.tif]
